# The interplay of BDNF-TrkB with NMDA receptor in propofol-induced cognition dysfunction: Mechanism for the effects of propofol on cognitive function

**DOI:** 10.1186/s12871-018-0491-y

**Published:** 2018-04-05

**Authors:** Junfei Zhou, Fang Wang, Jun Zhang, Jianfeng Li, Li Ma, Tieli Dong, Zhigang Zhuang

**Affiliations:** 1 Department of Pain, The Second Affiliated Hospital of Zhengzhou University, College of Medicine, No. 2 Jingba Road, Zhengzhou, 450003 China; 2 Department of Anesthesiology, The First Affiliated Hospital of Zhengzhou University, Zhengzhou, 450003 China; 3 Department of Anesthesiology, The Second Affiliated Hospital of Zhengzhou University, College of Medicine, No. 2 Jingba Road, Zhengzhou, 450003 China

**Keywords:** NMDA, BDNF, TrkB, Propofol, Cognition dysfunction

## Abstract

**Background:**

The aim of the present study was to verify whether propofol impaired learning and memory through the interplay of N-methyl-D-aspartate (NMDA) receptor with brain-derived neurotrophic factor (BDNF)-tyrosine kinase B (TrkB) signaling pathway.

**Methods:**

120 Sprague-Dawley (SD) rats were randomly assigned into eight groups. Experimental drugs including saline, intralipid, propofol, N-methyl-D-aspartate (NMDA), 7,8-dihydroxyflavone (7,8-DHF), K252a and MK-801. Spatial learning and memory of rats were tested by the Morris water maze (MWM) test. The mRNA and protein expression were determined by immunohistochemistry, RT-PCR and western blot. Finally, hippocampus cells proliferation and apoptosis were examined by PCNA immunohistochemistry and TUNEL respectively.

**Results:**

The memory and learning was diminished in the propofol exposure group, however, the impaired memory and learning of rats were improved with the addition of NMDA and 7,8-DHF, while the improvement of memory and learning of rats were reversed with the addition of K252a and MK-801. In addition, the mRNA and protein expression levels and hippocampus cells proliferation were the same trend with the results of the MWM test, while apoptosis in hippocampus was reversed.

**Conclusion:**

The propofol can impair memory and learning of rats and induce cognition dysfunction through the interplay of NMDA receptor and BDNF-TrkB-CREB signaling pathway.

## Background

General anesthesia is the most common procedure for surgery, particularly in children [1]. Previous studies suggested that anesthetic exposure can lead to neurotoxicity in the developing brain [2, 3]. Similarly, children exposed to anesthetics have been reported to exhibit a higher incidence of learning deficits [4]. The effects of general anesthesia on the developmental brain have drawn much attention from society. Propofol is a commonly used intravenous anesthetic, which is widely used in pediatric anesthesia [5]. However, a large number of cell experiments and animal studies have found that propofol can lead to cerebral neuronal apoptosis during development, and cause developmental abnormalities such as long-term learning and memory [6–8]. In addition, there are clinical reports of propofol-induced neurological dysfunction and behavioral abnormalities in children [9, 10]. Therefore, looking for safe and effective measures to prevent propofol-induced developmental brain damage has become a problem to be solved. Learning and memory is a very complex process, and the molecular and cellular mechanisms are still unclear. It has been known that learning and memory primary happens in the hippocampus [11]. Long-term potentiation (LTP), one of several phenomena underlying synaptic plasticity, is generally considered to be one of the primary cellular mechanisms involved in hippocampus-dependent learning and memory [12, 13]. However, activation of N-methyl-D-aspartate (NMDA) receptors at many excitatory synapses is required for activity-dependent induction of LTP [14], a cellular substrate for learning and memory. NMDA receptors can regulate glutamate secretion through elevating presynaptic Ca^2+^ signals [15], and subsequent the activation of CaM Kinase II, IV and MAPK following LTP induction conditions [16, 17]. Afterwards, CaM kinase IV and MAPK promote phosphorylation of CREB by co-stimulating gene expression, and then play a role in the LTP [18].

Brain-derived neurotrophic factor (BDNF), a cognate ligand for the tyrosine kinase receptor B (TrkB) receptor, is mainly synthesized by the brain and is distributed throughout the central nervous system, especially the hippocampus and cerebral cortex [19]. BDNF plays an important role in the neuronal survival, differentiation, synaptic plasticity and neurogenesis [20]. Mizuno et al. have determined that the acquisition of learning and memory is involved in an increase in BDNF mRNA expression in hippocampus [21]. Rats with BDNF defects showed impairments both in hippocampus-dependent learning and memory and hippocampus LTP [22].

At present, propofol is one of the most frequently used intravenous general anesthetics and is also used for pediatric anesthesia [23]. Some animal experiments have indicated that propofol anesthesia could induce neuronal apoptosis in hippocampus as well as cognitive deficits [24]. It is also reported that the damage of cognitive function caused by propofol in elderly patients [25]. However, the exact mechanism for the effects of propofol on cognitive function still needs further clarification.

In previous study, it can be found that propofol exposure impaired learning and memory of rats by disturbing the BDNF-TrkB signaling pathway [26], meanwhile, axonal NMDA receptors play a significant role in LTP induction by triggering activity-induced presynaptic secretion of BDNF [27]. Thereby, in our study, we aim to verify whether the interplay of BDNF-TrkB with NMDA receptor is involved in the propofol-induced cognition dysfunction in Hippocampus.

## Methods

### Management of experimental animals

120 Sprague-Dawley (SD) rats (Laboratory Animal Unit, Zhengzhou university, China), weighing 14 ± 2 g and 7 days of age were used in this study. The rats were weight-matched and then randomly assigned into eight groups (*n* = 15): control group (NS group) rats were intraperiotoneally administered with 100 mg/kg saline; intralipid group (I group) were given equal volumes of 10% intralipid; the other six groups: P group, PN group, PD group, PND group, PNK group and PMD group were intraperitoneally injected respectively with saline, NMDA, TrkB agonist 7,8-dihydroxyflavone (7,8-DHF), NMDA combination with 7,8-DHF, NMDA combination with TrkB antagonist K252a and NMDA receptor antagonist MK-801 combination with 7,8-DHF every day before 90 mg / kg of propofol was administered [28]. All experimental procedures in this study were approved by the Ethics Committee of The Second Affiliated Hospital of Zhengzhou University.

### Determination of cognitive function

Six rats of each group were selected randomly and raised to 40 days of age. Spatial learning and memory of them were tested by the MWM test. At the start of trial, each rat was gently put into the pool which filled with water at 24 ± 1 °C, facing the wall, at one of three randomized start positions, and then rat was allowed to escape onto the platform. The maze is placed in a room with a number of extra-maze cues and lights dimmed on the walls that the rat can use to navigate the maze. Rats were given two times per day for five consecutive days of training in the Morris water maze. The time to find the platform for the rat (which indicated learning ability) was recorded as the escape latency. The rat was allowed 60 s to escape onto the platform; if the rat failed to find the platform within 2 min, it was guided to the platform and remained there for 10 s. At the end of each trial, rats were towel-dried, returned to its cage for approximately 15–20 min before its next trial. On day 7, the platform was removed; a reversal test was performed for 5 trials for the rats, followed by a probe trial for 120 s, in order to evaluate the capacity of the rats to relearn a new platform location. Performance was recorded and analyzed with a video connected to a computer, which allows measurements of latency of finding the platform, path length and swimming speed, as well as information on number of platform-crossings and time spent in the target quadrant (where the platform had been previously located) during the probe trials. The mean value of the latencies, target quadrant times or platform-crossing times of the rats were calculated as the final results [29].

### Expression of NMDA receptor, proBDNF, mBDNF, TrkB, ERK1 and CREB in hippocampus

Rats were anaesthetized and killed by cervical dislocation after the MWM test. Hippocampus tissues were harvested and divided into several sections, afterwards, immersed in 4% paraformaldehyde for immunohistochemistry or stored at − 80 °C for RT-PCR and western blot analyses. A part of hippocampus tissues were embedded in paraffin, and cut into 5 μm thickness serial sections [26]. After deparaffinization, rehydration and antigen retrieval, tissue sections were incubated with the rabbit polyclonal anti-NMDA receptor (NMDAR) antibody (PA1222, BosterBio) and rabbit polyclonal anti-BDNF antibody (D121057, Sangon Biotech, China) at 37 °C for 1 h. After cultured with the relevant secondary antibody, the results were observed by an optical microscope. The mRNA expression of NMDA receptor, proBDNF, mBDNF, TrkB, ERK1 and CREB were determined by RT-PCR. Total RNA was extracted from another part of hippocampus tissues with the TRIzol kit (Takara). cDNA synthesis was completed according to the manufacturer’s instructions and Real-time PCR was conducted by using the SYBR Green PCR Kit. Total protein lysates were prepared by the other part of homogenizing hippocampal tissues in lysis buffer (Thermo Scientific, USA). The protein expression and phosphorylation of NMDA receptor, proBDNF, mBDNF, TrkB, ERK1 and CREB were determined by Western blot using specific monoclonal antibodies as follows: rabbit anti-NMDAR (PA1222, BosterBio), rabbit anti-p-NMDAR (ABN99, Millipore), mouse anti-proBDNF (sc-65,514, Santa Cruz), rabbit anti-mBDNF (D160119, Sangon Biotech, China), rabbit anti-TrkB (ab18987, Abcam), rabbit anti-p-TrkB (ABN1381, Millipore), rabbit anti-CREB (ab32515, Abacm), rabbit anti-p-CREB (ab32096, Abcam).

### Determination of hippocampus cells proliferation and apoptosis

The hippocampus cells proliferation were performed by PCNA immunohistochemistry, in brief, tissue sections were incubated with PCNA mouse monoclonal antibody (0.2 μg/ml, AF0261, Beyotime, China) for 45 min and the results were observed by an optical microscope [30]. Meanwhile, hippocampus tissue sections were incubated with TUNEL reagent (Roche, Germany) for 60 min at 37 °C, and then stained with Hoechst 33,258 (1 μg/ml, Sigma-Aldrich, USA) for 20 min [31]. Afterward, the tissue sections were mounted with mounting medium (Applygen Technologies Inc., China) and visualized under a confocal microscope. Five visual fields were randomly selected, and the percentage of positive hippocampal cells was calculated as the apoptosis index.

## Results

### The ameliorating effects of NMDA and DHF in impaired learning and memory of rats

**Fig. 1:**
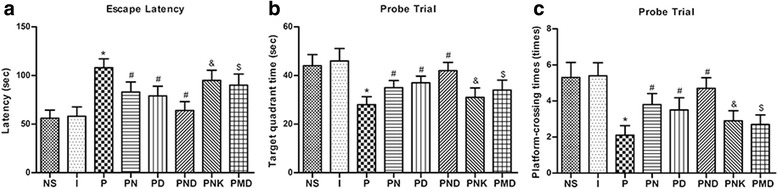
The learning and memory of 40-day-old rats were tested by Morris water maze (MWM). A. The rats in required different time (escape latency) to find the hidden the platform in different groups. B. Target quadrant travelling time of different groups. C. Platform-crossing times of different groups. *versus NS group, *P* < 0.05; #versus P group, P < 0.05; &versus PN group, P < 0.05; $versus PD group, P < 0.05

Spatial learning and memory of the rats was tested with MWM analysis 6 h after drug treatment. Results revealed that compared to the control group, there was no significant difference of learning and memory in the 10% intralipid group (I group). This is consistent with previous reports [26]. Compared to the NS group, the rats in the propofol exposure group (P group) had increased latencies to find the hidden platform but had shorter platform-crossing times and shorter target travelling time. These results suggest that exposure to propofol impairs learning and memory in rats. Interestingly, the results showed that the deficits were improved in the PN, PD and PND group in comparison with those in the P group, while the learning and memory in the PND group was better than those in the PN and PD group. These results indicate that the NMDA receptor agonist NMDA and TrkB agonist 7,8-DHF can obviously improve the impaired learning and memory induced by propofol but cannot completely reverse these impairments. Additionally, compared with PN group, the improvement effect of the PNK group was inhibited; similarly, the improvement effect of the PMD group was inhibited in comparison with PD group. All these results indicated that the NMDA and DHF could ameliorate impaired learning and memory of rats, besides, the interaction of BDNF-TrkB and NMDA receptor might have effects on cognitive function (Fig. 1).

### The expression of NMDAR and BDNF in immunohistochemistry

To determine whether NMDA receptor and BDNF are involved in the learning and memory impairments observed following rats propofol exposure, the protein levels of NMDA receptor, and BDNF in hippocampus tissues of the rats were detected. Immunohistochemistry staining showed that there were no significant difference between PN group and PD group. PNK and PMD group also showed a similar effect. However, NMDA receptor and BDNF were expressed abundantly both in the nucleus and cytoplasm in the PND group in hippocampus. Specifically, the protein levels of NMDA receptor and BDNF in the P group were significant less than those in the NS group, while up-regulated expression of NMDAR and BDNF in the PN, PD and PND group compared to P group. Among them, the PND group had the highest expression level. What’s more, the improvement effects of the PNK or PMD group were inhibited compared to PN or PD group. These results suggest that the observed impairments in learning and memory may be related to the decreased protein levels of NMDAR and BDNF.

### Expression of NMDAR, proBDNF, mBDNF, TrkB, ERK1 and CREB

**Fig. 2:**
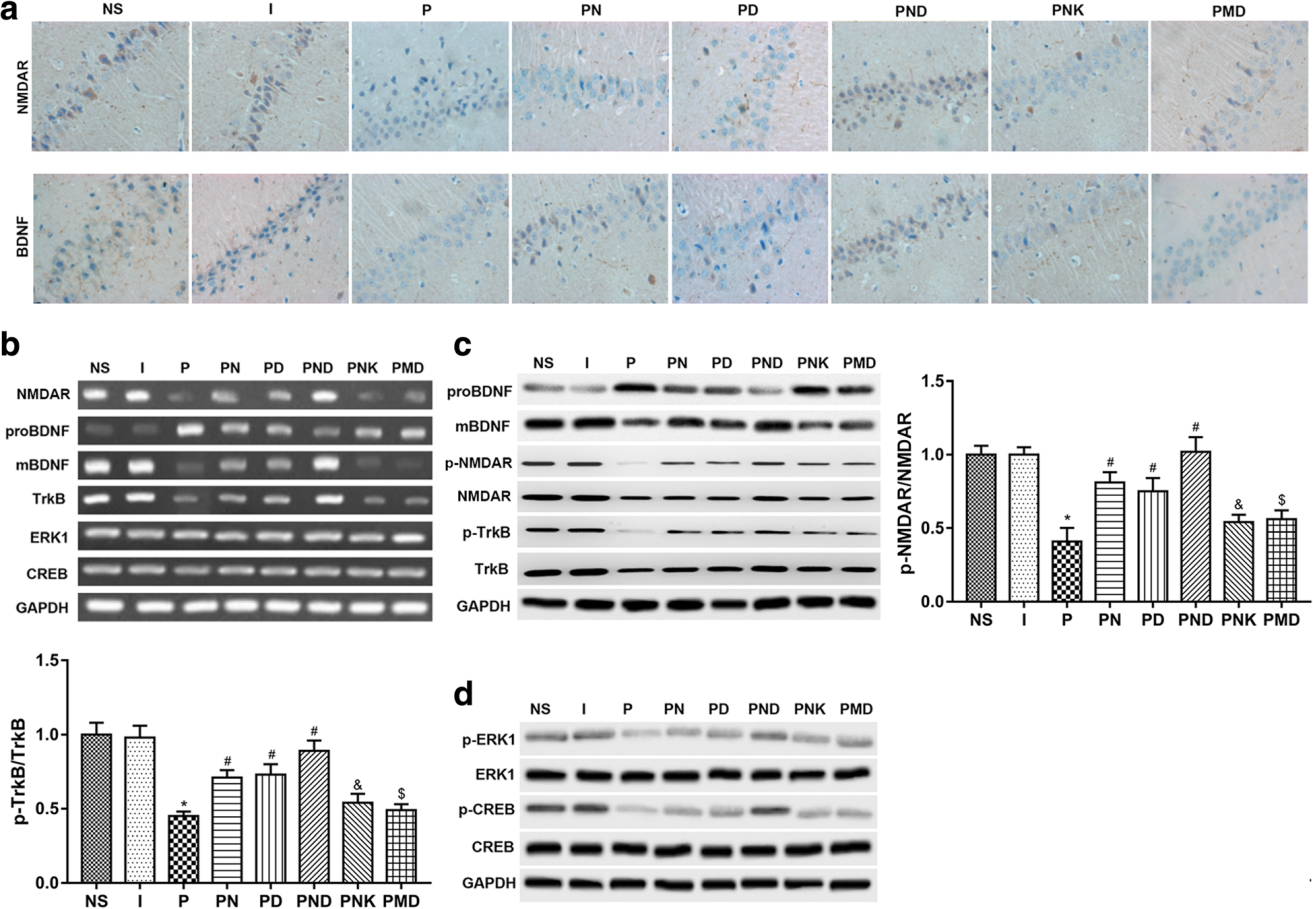
Immunohistochemical staining revealed that the protein levels of NMDAR and BDNF in hippocampus tissue. A. NMDAR and BDNF were expressed abundantly both in the nucleus and cytoplasm in the PND group, the improvement effect of the PNK group was inhibited compared to PN, the improvement effect of the PMD group was inhibited compared PD group. B. The mRNA expression of NMDAR, proBDNF, mBDNF, TrkB, ERK1 and CREB were tested by RT-PCR. The mRNA levels of NMDAR, mBDNF and TrkB were lower in the P group than those in the NS group, while the level of proBDNF was up-regulated in the P group compared to NS group. However, there were no obvious different expression of ERK1and CREB between the NS group and the others. C, F. The protein phosphorylation of NMDAR, proBDNF, mBDNF, TrkB, ERK1 and CREB were determined by Western blot. The phosphorylation levels of NMDAR, TrkB, ERK1 and CREB in the PND group were highest, while P group were the lowest. D, E. The ratio of p-NMDAR and NMDAR, the ratio of p-TrkB andTrkB. *versus NS group, P < 0.05; #versus P group, P < 0.05; &versus PN group, P < 0.05; $versus PD group, P < 0.05

As shown in Fig. 2B, the mRNA levels of NMDAR, mBDNF and TrkB were lower in the P group than those in the NS group, while the level of proBDNF was up-regulated in the P group compared to NS group. These results reveal that the NMDAR agonist and TrkB agonist (NMDA and 7,8-DHF) can reverse the down-regulation of NMDAR, BDNF and TrkB caused by propofol exposure but cannot alleviate the impaired effects completely. As showed in Fig. 2C-F, the trend of protein expression was similar to the mRNA expression, suggesting that down-regulated mRNA expression is involved in the decrease of protein abundance induced by propofol exposure. Interestingly, the phosphorylation levels of NMDA receptor, TrkB, ERK1 and CREB in the PND group were higher than those in the other group, while the phosphorylation levels in the P group were lowest. These suggested that NMDAR and BDNF-TrkB might be involved in the cognition through the BDNF-ERK1-CREB signaling pathway.

### Hippocampus cells proliferation and apoptosis

**Fig. 3:**
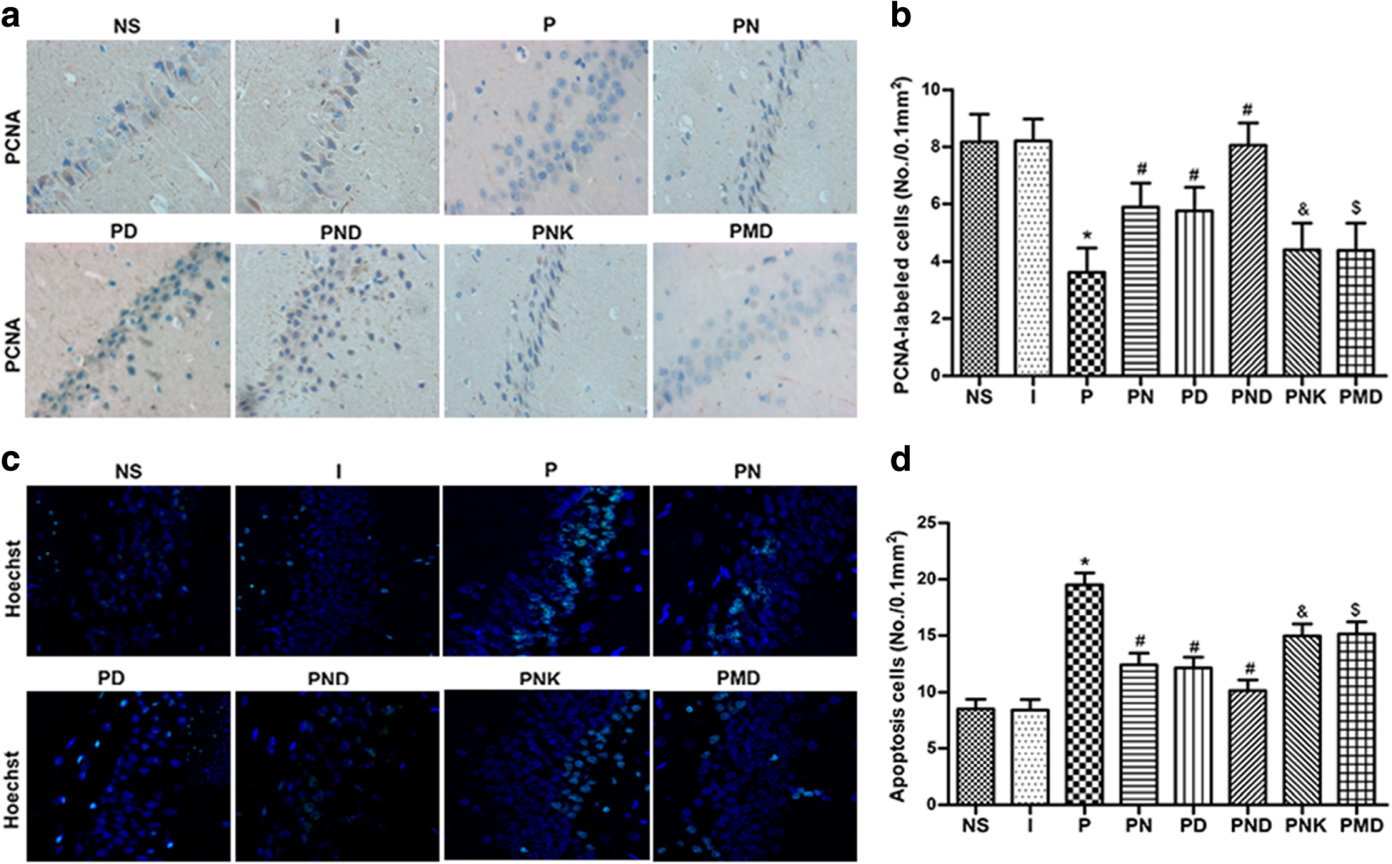
Hippocampus cells proliferation were detected by PCNA immunehistochemistry and apoptosis were detected with TUNEL. A. Compared to the NS group, the hippocampus cells proliferation was obviously decreased in the P group, the hippocampus cells proliferation were increased in the PN, PD and PND group. B. Apoptosis in hippocampus were increased in the P group and were decreased in the PN, PD and PND group. *versus NS group, P < 0.05; #versus P group, P < 0.05; & versus PN group, P < 0.05; $versus PD group, P < 0.05

To further verify whether the NMDAR and BDNF-TrkB signaling pathway are involved in cognition (learning and memory), PCNA immunohistochemistry and TUNEL were performed. The results shown in Fig. 3, compared to the NS group, the hippocampus cells proliferation were obviously decreased in the P group, and compared to the PN and PD group, the hippocampus cells proliferation were significantly increased in the PND group. However, apoptosis in hippocampus were increased in the P group in comparison with NS group while decreased in the PND group in comparison with compared to PN and PD group. These results confirm that the learning and memory impairments induced by propofol exposure correlate with the interplay of NMDAR and BDNF-TrkB signaling pathway.

## Discussion

It is known that the most frequently used general anesthetics have either NMDA receptor blocking or γ-aminobutyric acid (GABA) receptor activating properties in regulating neurodegeneration and cognition dysfunction in the developing brain. In particular, many intravenous anesthetics as well as inhalational volatile anesthetics promote inhibitory neurotransmission by enhancing GABA-induced currents in neuronal tissue. For this reason, they are often referred to as GABAergic agents [32]. Propofol has been proved to be a more appropriate anaesthetic by enhancing GABAergic transmission [33]. Previous studies also have shown that propofol may also block NMDA receptor via an inhibitory effect on NR1 subunit phosphorylation in neurons [34]. Another experiment in cultured hippocampal neurones showed that propofol causes a reversible inhibition of NMDA receptors through reducing the frequency of NMDA-activated single channel openings [35]. Propofol may impair learning and memory by preventing synaptic plasticity (especially LTP) [36], and by inhibiting NMDA and AMPA receptors [37], as well as by inhibiting 5-HT receptors [38] and nicotinic acetylcholine [39]. Some studies have indicated that propofol may facilitate the maintenance of LTD in the hippocampus [36]. Together, these researches suggest that propofol can impair learning and memory by affecting the expression of multiple genes and altering proteins interactions. Brain derived neurotrophic factor (BDNF) was first researched by German neurobiologist Barde in 1982 [40]. BDNF promotes Tyrosine kinase autophosphorylation and dimerization by binding to the Tyrosine kinase B (TrkB) receptor and activating intracellular signal transduction pathways. BDNF also plays a vital role in regulating the plasticity of synapses through activating TrkB receptor [19, 20]. Some reports showed that activation of NMDA receptor in postsynaptic dendrites is required for long-term potentiation (LTP) of many excitatory synapses [14], besides, axonal NMDA receptor play an important role in LTP induction at corticostriatal synapses by triggering activity-induced presynaptic secretion of BDNF [27]. In the rats experiment, the results showed that the learning and memory was diminished in the propofol exposure group. However, with the addition of NMDA and 7,8-DHF (NMDA receptor agonist and TrkB agonist), the inhibition of learning and memory of rats were improved, suggesting that the NMDA receptor and BDNF-TrkB signaling pathway may play a significant role in propofol-induced learning and memory impairment.

Moreover, it can be seen that the protein expression levels of NMDA receptor and BDNF were lower in the propofol exposure group than control group in hippocampus by immunohistochemistry, meanwhile, the mRNA and protein expression levels of NMDA receptor, mBDNF and TrkB were the same trend in the P group. However, NMDA receptor and TrkB agonists could reverse the inhibition. These verified the previous point that the interplay of BDNF-TrkB with NMDA receptor was involved in the propofol-induced cognition dysfunction in hippocampus. Additionally, the results showed that phosphorylation levels of NMDA receptor, TrkB, ERK1 and CREB were lower in the P group while up-regulated in the PND group with addition of NMDA receptor and TrkB agonists, suggesting the propofol may impair learning and memory of rats through inhibiting NMDA receptor and BDNF-TrkB signaling pathway. Sung W et al. said that TrkB receptors can activate the downstream signaling cascade by binds with high affinity to TrkB agonist 7,8-DHF [41], thereby improving learning and memory.

It has been found that presynaptic NMDA receptors are found in various brain tissues, it can regulate glutamate release and trigger presynaptic BDNF secretion via elevating presynaptic Ca^2+^ signals [42–45]. TrkB, a cognate receptor for the BDNF, mediates neuronal differentiation, survival, synaptic plasticity, and neurogenesis [46]. However, 7,8-DHF as a bioactive high-affinity TrkB agonist that provokes receptor dimerization and autophosphorylation and protected neurons from apoptosis by regulating downstream signaling including PI3K/Akt [47] and MAPK [48], Erk1/2 and ERKB [41]. Interestingly, in our study, it can be found that phosphorylation levels of ERK1 and CREB were down-regulated, the hippocampus cells proliferation were decreased and apoptosis were increased in the P group, indicating that the propofol impair learning and memory of rats via inhibiting BDNF-TrkB-CREB signaling pathway.

## Conclusion

Consequently, the propofol can impair memory and learning of rats and induce cognition dysfunction through the interplay of NMDA receptor and BDNF-TrkB-CREB signaling pathway.

## Abbreviations

7,8-DHF: 7,8-dihydroxyflavone
AMPA: α-amino-3-hydroxy-5-methyl-4-isoxazolepropionic acid
BDNF: Brain-derived neurotrophic factor
CREB: cAMP-response element binding protein
ERK: Extracellular regulated protein kinases
LTP: Long term potentiation
MAPK: Mitogen-activated protein kinase
MWM: Morris water maze.
NMDA: N-methyl-D-aspartate.
POCD: Postoperative cognitive dysfunction.
SD: Sprague-Dawley.
TrkB: Tyrosine kinase receptor B.
